# Preparation of Surface Adsorbed and Impregnated Multi-walled Carbon Nanotube/Nylon-6 Nanofiber Composites and Investigation of their Gas Sensing Ability

**DOI:** 10.3390/s90100086

**Published:** 2009-01-05

**Authors:** Neeta L. Lala, Velmurugan Thavasi, Seeram Ramakrishna

**Affiliations:** 1 Department of Mechanical Engineering, National University of Singapore, 2 Engineering Drive 1, Singapore 117576; E-Mail: mpenll@nus.edu.sg; 2 Nanoscience and Nanotechnology Initiative, National University of Singapore, 2 Engineering Drive 3, Singapore 117576; E-Mail: nnitv@nus.edu.sg; 3 Division of Bioengineering, National University of Singapore, 9 Engineering Drive 1, Singapore 117576

**Keywords:** Porosity, electrospinning, nanofibers, dispersion, one dimensional material, surface functionalization

## Abstract

We have prepared electrospun Nylon-6 nanofibers via electrospinning, and adsorbed multi-walled carbon nanotubes (MWCNTs) onto the surface of Nylon-6 fibers using Triton^®^ X-100 to form a MWCNTs/Nylon-6 nanofiber composite. The dispersed MWCNTs have been found to be stable in hexafluoroisopropanol for several months without precipitation. A MWCNTs/Nylon-6 nanofiber composite based chemical sensor has demonstrated its responsiveness towards a wide range of solvent vapours at room temperature and only mg quantities of MWCNTs were expended. The large surface area and porous nature of the electrospun Nylon-6/MWCNT nanofibers facilitates greater analyte permeability. The experimental analysis has indicated that the dipole moment, functional group and vapour pressure of the analytes determine the magnitude of the responsiveness.

## 1. Introduction

Sensors that can detect gaseous molecules in industrial, medical, and living environments are in great demand nowadays [1-6]. Higher responsiveness, greater selectivity and shorter response times have therefore been the key requirements for gas sensors. Selection of materials and their interfaces have been central to meeting such requirements. Sensors based on conducting polymers have attracted attention as they have the potential for better processability and cost effectiveness [7], however they typically suffer from thermal and environmental stability issues, which prevent their scalability for commercial applications. Non-conducting polymers (insulators) possess relatively better stability and easy processability and hence are considered as potential alternate sensor material, provided the electrical conductivity can be induced. Research activities have thus been intensified on tailoring the electrical property of non-conducting polymer composites by loading with electrically active fillers [8-10].

Fillers have been successful in imparting electrical conductivity to non-conducting polymers, but the conductivity is dependent on the type of filler used [11]. Moreover, fillers are susceptible to mechanical or structural degradation if not selected properly, for instance, a higher loading of carbon black (CB) results in an increase in viscosity and consequently, impairment in the polymer properties [12]. The higher aspect ratio of carbon nanotubes (CNTs) has been promising because it imparts not only electrical conductivity to insulators, but also at lower loadings compared to CB and carbon fibers [13-16]. Multi-walled carbon nanotubes (MWCNTs) are more robust in structure, they display higher rigidity and are cheaper than SWCNTs, which are advantageous for their use as sensing probe materials [17, 18]. Furthermore, MWCNTs provide a novel electrode platform and good operability for sensors that operate at room temperature. MWCNTs have been employed in this study as the conductive fillers. Nylon-6, an insulating polymer known for its excellent compatibility [19] has been chosen as the host matrix for the MWCNTs in this study.

Firstly, aggregation and poor interfacial interaction are the major problems encountered when conductive fillers are used in a host polymer matrix [20-21]. Aggregation could be minimized if the host matrix has a one dimensional (1-D) morphology as this provides structural integrity for better dispersion. Given the advantage of a larger surface area through 1-D morphology, the response of the sensor can be improved [22-23]. A recent study has suggested that the aggregated MWCNTs limit the analyte interaction sites and this result in poor detection of gas analytes [24]. The host matrix should therefore facilitate better interfacial contact with the fillers (MWCNTs) and simultaneously allow for analyte permeability, which are possible in 1-D nanofiber structures because of their porous structure. Our recent review has highlighted that 1-D electrospun nanofibers are preferable for energy and electronic devices since the electron flow is focused, leading to enhanced sensitivity [25]. Electrospinning (ES) has been one of the most versatile and cost-effective methodologies to produce 1-D nanofiber materials [26] and researchers have proposed electrospun nanofibers for the fabrication of lower cost and rapid response sensors [27-28].

Secondly, unless the MWCNTs are uniformly dispersed in the nylon-6 matrix, the full advantage of the electronic properties of individual MWCNTs will not be realized. Researchers have developed methods to disperse the CNTs, such as functionalizing CNTs on the polymer [29], solution casting [30], dip-coating [31-32], layer by layer (LBL) assembly [33], high-energy sonication [34], wet spinning and surfactant-assisted processing [35-36]. However, methods such as solution casting [37] could lead to aggregation of CNTs during solvent evaporation. Likewise, the chemical functionalization of CNTs may deteriorate the polymer's mechanical properties, and also of the ends and sidewalls of the CNTs [38]. Functionalization may also influence the reversibility of a sensor because of the possible exertion of stronger bonding with the analyte molecule.

In this study, the MWCNTs/Nylon-6 nanofiber composites have been prepared by adsorbing the MWCNTs on the surface of Nylon-6 nanofibers with the aid of Triton^®^ X-100 as surfactant and also impregnating (*im*) them within the Nylon-6 nanofibers. Their performance for chemical gas sensing has then been investigated.

## 2. Experimental

### 2.1. Materials

Nylon-6 polymer (average molecular weight of 13,000 -15,000) was obtained from UBE Industries Ltd (Japan). Hexafluoroisopropanol (HFIP, 99%), *N,N*,-dimethylformamide (DMF), methanol, acetone, and trichloromethane have been purchased from Sigma-Aldrich (Germany). *N,N*-dimethylacetamide (DMA), tetrahydrofuran (THF), ethyl acetate (EA), dichloromethane (DCM), trichloromethane (TCM), hexane and toluene have been procured from Merck (Darmstadt). Ethanol has been bought from Fisher Scientific (UK). MWCNTs (95% purity), each of 10-30 nm in diameter, 1-2 μm in length, 40-300 m^2^/g of surface-area, have been obtained from Shenzhen Nanotech Port Co Ltd, (P.R. China).

### 2.2. Methods

**Figure 1.**
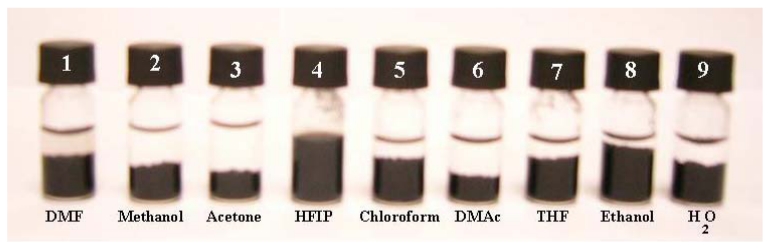
Images of vials taken one month after the dispersion of 7.5 mg of MWCNTs in 2 mL of various solvents. The MWCNTs dispersed in HFIP (bottle number 4) has demonstrated best dispersion and greater stability.

The dispersibility of MWCNTs has been examined in various solvents: dimethylformamide (DMF), methanol, acetone, HFIP, trichloromethane, DMA, THF, ethanol and water. HFIP has been found to be the best solvent for dispersing the MWCNTs effectively. The solution has also been found to be stable at room temperature for over several months without precipitation (Figure 1).

Nylon-6 polymer was dissolved in HFIP solvent to form the Nylon-6 solution for electrospinning. Microscopic glass-slides were cleaned before use and then cut to a size of 25 × 20 mm. Gold as the electrode was sputtered on an area of 25 × 3 mm size on the two opposite ends of glass-slide for the electrical connection. Then the glass-slide has been carefully masked with paraffin film so that a blank space of 10 × 20 mm area is available for the deposition of electrospun polymer nanofibers.

#### 2.2.1. Nylon-6 nanofibers

**Figure 2.**
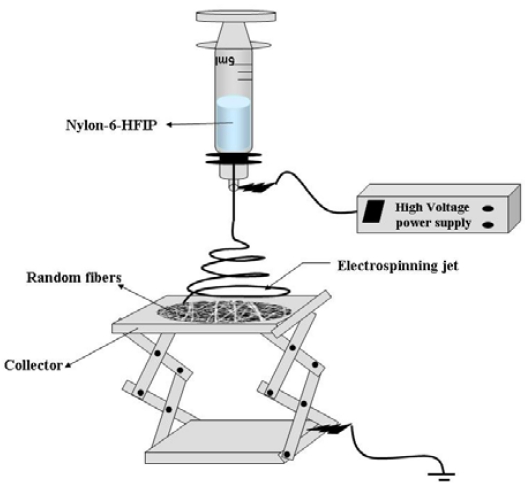
Schematic of the electrospinning process to obtain the Nylon-6 nanofiber mat. Solution of 7 wt% Nylon-6 in HFIP has been electrospun by feeding through a syringe pump, and the feed rate has been set at 2 mL/h. The metallic needle of the syringe has been connected to a high-voltage power supply, and the glass-slide has been placed 10 cm below the tip of the needle to collect the nanofibers.

The electrospinning setup has possessed a high-voltage power supply, a syringe pump, a syringe, and a grounded collector (Figure 2). Nylon-6 polymer (0.7 g) was dissolved in HFIP solvent (9.3 g) and stirred overnight. The role of HFIP is therefore to dissolve Nylon-6 polymer. The resulting Nylon-6 polymer solution was transferred into a 5 mL volume syringe (0.859 mm in diameter) and positioned in the dispenser pump. The metallic needle of syringe has been connected to a high-voltage power supply. The previously prepared gold sputtered glass-slide was placed on the collector stand at a distance of about 10 cm below the tip of the needle to collect the composite nanofibers directly on the glass-slide. The solution feed rate was set at 2 mL/h. Upon applying a voltage of 15 kV to the needle of the syringe, a jet was formed at the end of the syringe needle, resulting in the formation of Nylon-6 nanofibers during the deposition on the glass-slide. The nanofibers have been collected continuously until a mat of the nanofiber composite with a thickness of approximately 0.3 mm was formed. Then the nanofiber composite thus prepared was dried under vacuum overnight in air at 50°C.

**Figure 3.**
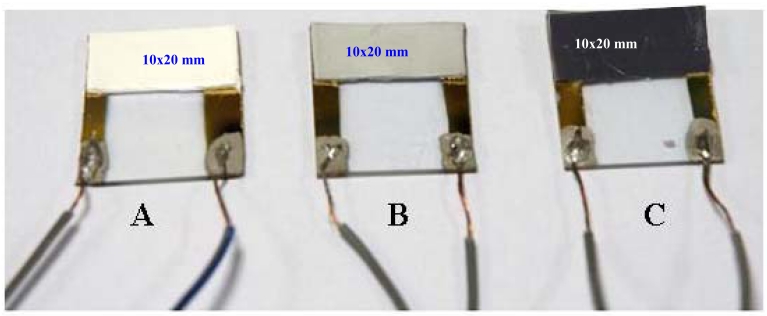
Sensing device fabricated with (A) neat Nylon-6 nanofibers on an area of 10 × 20 mm as control and (B) *im* MWCNTs/Nylon-6 nanofibers on an area of 10 × 20 mm; (C) *sa* MWCNT on Nylon-6 nanofibers of 10 × 20 mm area. Gold has been used as counter electrode on the two opposite ends of the glass-slide on an area of 25 × 3 mm size.

The prepared Nylon-6 nanofiber composite mat was immersed in a bath containing dispersed MWCNTs (5 mg) and investigated in different concentrations (0.1, 0.3, 0.5, 1 and 2 wt %) of Triton X^®^-100 surfactant solution. The entire setup has been kept on a shaker (Orbital shaker SO3, UK) at ∼75 rpm for a period of 1 min in order to allow for uniform adsorption of MWCNTs on the surface of the Nylon-6 nanofiber composite adhered-slide. A uniform dispersion of MWCNTs on the surface of the Nylon-6 nanofibers was achieved for the 1 wt % concentration of Triton^®^ X-100 surfactant. The adhered-nanofiber composite slide was then rinsed with de-ionized water and sonicated repeatedly until the excess of surfactant, as well as the loosely bound MWCNTs are removed. As a result, the *sa* MWCNT/ Nylon-6 nanofibers mat (Figure 3B) was obtained and then investigated for sensing performance of the device.

The study has also been designed to examine impregnation (*im*) of the MWCNTs inside the Nylon-6 polymer nanofibers in order to compare the sensing performance with the *sa* MWCNT/Nylon-6 nanofibers composite mat. A 1 wt % MWCNTs and Nylon-6 polymer/HFIP solution were blended using an ultrasonicator. The resulting mixture was electrospun, keeping the same above mentioned electrospinning conditions to form *im* MWCNTs/Nylon-6 nanofibers. It should be mentioned that for *im* MWCNTs/Nylon-6 nanofibers, the Triton^®^ X-100 surfactant was not used whereas for the preparation of *sa* MWCNTs/Nylon-6 nanofibers it was used because the role of surfactant is critical for the adsorption of MWCNTs onto the surface of Nylon-6 nanofibers. The presence of surfactant not only prevents the MWCNTs from aggregating, which results from a substantial van der Waals interaction between MWCNTs, but also induces the interaction of MWCNTs and Nylon-6 polymer that causes them to adhere on the surface and self assemble. The Triton^®^-X surfactant was not employed in the preparation of *im* MWCNTs/Nylon-6 nanofibers because the use of surfactant would lead to nanofiber pore blockage and act as a barrier for the effective interaction of analytes and the MWCNTs/Nylon-6 nanofibers. The adhered-*im* MWCNT/Nylon-6 nanofiber slide (Figure 3C) has been also examined for sensing performance.

**Figure 4.**
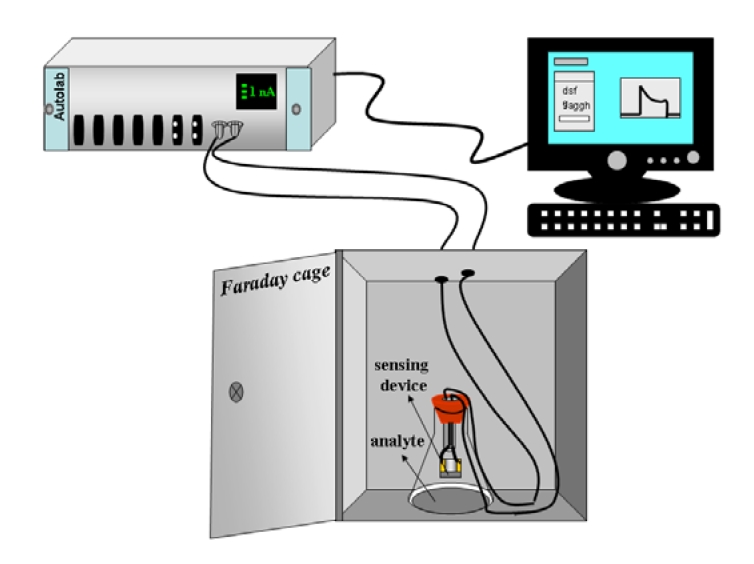
Schematic of sensor set up. The sensing device made up of MWCNTs/Nylon-6 nanofibers composite has been attached in a conical flask with a rubber stopper and then connected to the Autolab (model PGSTAT 30). The sensing set up has been enclosed in a Faraday cage to ensure a protected environment.

The experimental set up has been constructed as follows: high purity analyte (solvent, 3 mL) was placed in a conical glass flask, and sealed with a two holed rubber stopper. The sensing device was attached (Figure 4). The distance between the sensing device and the solvent surface level has been kept constant (12 cm) throughout the experiments. Extra care has been taken to prevent the device from being exposed to the external atmosphere.

## 3. Results and Discussion

The *sa* MWCNTs/Nylon-6 nanofibers have been characterized using a Quanta 200F scanning electron microscope (SEM) which operates at 10 kV, whereas the *im* MWCNTs/Nylon-6 nanofibers have been examined using a JEM 2010F transmission electron microscope (TEM) operated at an accelerating voltage of 200 kV.

**Figure 5.**
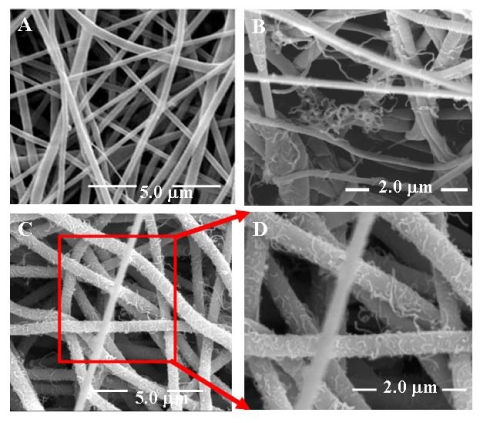
SEM images of (A) neat Nylon-6 nanofibers; (B) cross-sectional view of *sa* MWCNTs/Nylon-6 nanofiber composite showing that the MWCNTs reach even inside the electrospun nanofiber mat; (C) *sa* MWCNTs on the Nylon-6 nanofibers; (D) enlarged view of *sa* MWCNTs on Nylon-6 nanofibers.

Examination under SEM has shown that the average diameter of nanofibers is in the range of 110 - 140 nm (Figure 5A-D). Recently Kim *et al.* [39] have demonstrated the grafting of MWCNTs on the surface of electrospun poly(2-hydroxyethyl methacrylate (PMMA) nanofibers using Triton^®^ X-100 as surfactant, however they encountered blockage of pores by the MWCNTs that resulted in a lower responsiveness. Pore blockage has been prevented in our study by optimizing the electrospinning conditions used to obtain nanofibers of Nylon-6 polymer of well-defined 1-D nanostructures and high porosity. Cross-sectional examination of our MWCNTs/Nylon-6 nanofiber composite mat using SEM (Figure 5B) has revealed that the MWCNTs penetrate even the inner part of composite mat and adsorb on the nanofibers. This is due to the porous matrix, which clearly highlights the advantages of the electrospinning technique. The SEM image of *sa* MWCNTs/Nylon-6 nanofibers shows that the MWCNTs are interconnected to each other (Figures 5C and D).

**Figure 6.**
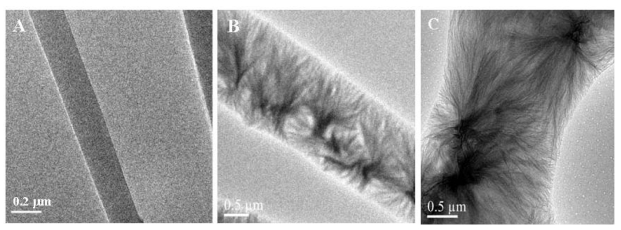
TEM images of (A) neat Nylon-6 nanofibers; (B) and (C) *im* MWCNTs within the electrospun nanofibers of 7 wt % Nylon-6.

The TEM images of *im* MWCNTs/Nylon-6 nanofibers also show that the MWCNTs are interlinked inside the Nylon-6 nanofibers (Figures 6B and C). The superior dispersion of MWCNTs on Nylon-6 strengthens the interconnectivity, not only between the MWCNTs, but also between the MWCNTs and Nylon-6 nanofibers. Researchers have observed that the MWCNTs interacts with the peptide groups of the silk nanofibers and strengthen the adsorption of MWCNTs on the surface of silk [40-43].

Nylon-6, a polymer that possesses a repeating unit of amide and carbonyl groups in its structure could interact with MWCNTs upon surface adsorption through hydrogen bonding. An increase in the interconnectedness of the MWCNTs on the surface of Nylon-6 nanofibers, in turn provides a continuous conducting path, known as ‘*percolation threshold* [44] and thus, induces the charge transport in MWCNTs. Hence, in principle the charge transfer takes place from the MWCNTs to Nylon-6 through the carbonyl (C=O) groups of the latter, resulting in continuous electron transport in the Nylon-6 composite matrix. The *sa* MWCNTs/Nylon-6 nanofiber composite based device was exposed at room temperature to wide variety of organic analytes, listed in Table 1. During the exposure to analytes, no measurable current signal has been observed for the sensing device that was constructed with Nylon-6 nanofibers alone. For the device that has been based on *sa* MWCNTs/Nylon-6 nanofiber composite, the responsiveness (*S*) towards various analytes has been calculated using the equations 1 and 2 [45], and the results are presented in Table 1.

(1) $$\text{Sensitivity},S=\frac{R-R_{0}}{R_{0}}=\frac{\frac{V}{I}-\frac{V_{0}}{I_{0}}}{\frac{V_{0}}{I_{0}}}$$

where *R_0_* and *I_0_* are the initial resistance and current at time t = 0 and *R* and I are the resistance and current obtained at maximum steady state value. For 1 volt, the above equation transformed as follows:

(2) $$S=\frac{I_{0}-I}{I}$$

**Figure 7.**
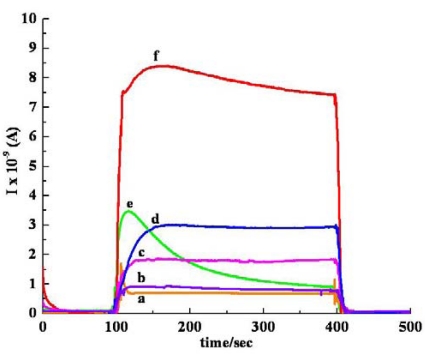
Response profile obtained for all analytes by *sa* MWCNT/Nylon-6 nanofibers at room temperature: (a) trichloromethane, (b) THF (c) EA, (d) ethanol, (e) acetone, (f) DCM.

All the experiments have been run in triplicate and it was observed that the overall variations of the responsiveness are within 10%. Figure 7, which shows the comparative response of *sa* MWCNTs/Nylon-6 nanofiber composite towards various polar organic analytes, clearly indicates that the nanofiber composite is responding to the various type of analytes used.

**Figure 8.**
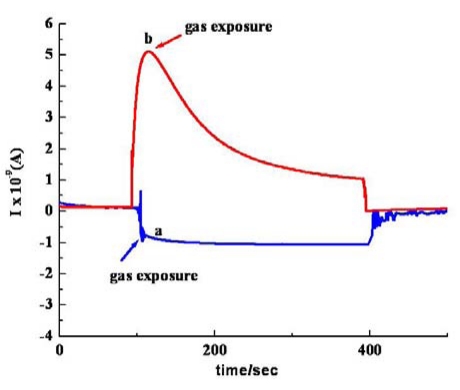
Response profile obtained at room temperature for (a) neat Nylon nanofibers based device, (b) *sa* MWCNT/Nylon-6 nanofibers based device when exposed to acetone vapour.

Figure 8 compares the responsiveness of the *sa* MWCNTs/Nylon-6 nanofiber based device and neat Nylon-6 nanofiber based device (control) towards acetone vapor. In the current work, all the analyte molecules have been involved in a small level of charge transfer, suggesting a weaker interaction between analyte and MWCNTs. Desorption of analyte molecules from the sensor device has been carried out by keeping the device open under ambient air conditions overnight and at room temperature. The reversibility has been checked and confirmed for three repeated measurement cycles, indicating that the detachment of gas molecules is possible at room temperature because of the weaker interaction between MWCNTs and analyte molecules.

**Figure 9.**
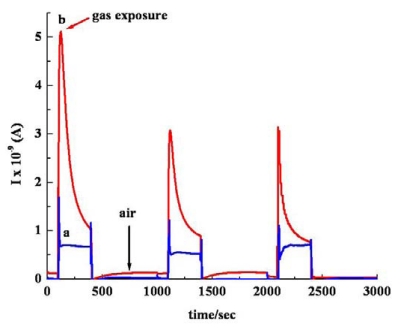
Reversibility of *sa* MWCNTs/Nylon-6 nanofibers when exposed to solvents: (a) acetone, (b) trichloromethane at room temperature.

**Figure 10.**
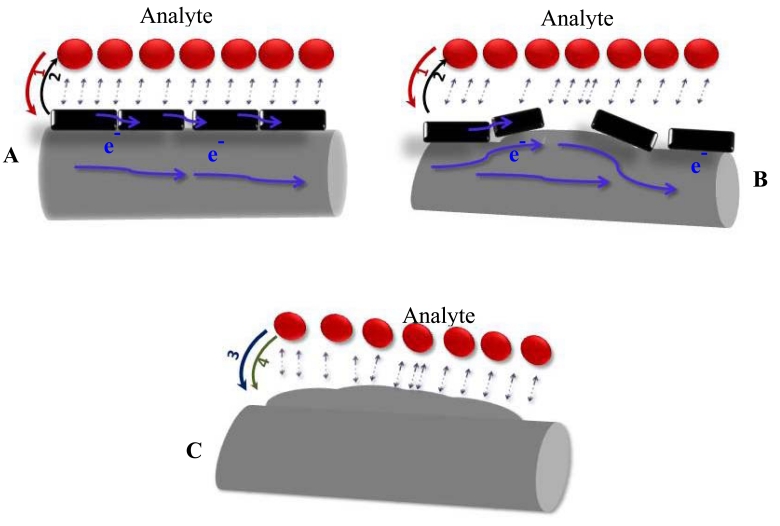
Three possible modes of analyte interactions: (A) Analyte exposure to MWCNTs directly; (B) Partial exposure of analyte to Nylon-6 and MWCNTs; (C) Analyte exposure to Nylon-6 polymer directly. Possible predominant mechanism: (1) electron transfers from analyte to MWCNTs if analyte is electron donor; (2) electron transfers from MWCNTs to analyte if analyte is electron acceptor; (3) electron transfers from analyte to polymer; (4) physical adsorption of analyte on Nylon-6 polymer that causes swelling.

The reusability and stability of our *sa* MWCNTs/Nylon-6 nanofiber based gas sensor has shown promise during the repeated tests, which are shown in Figure 9. The obtained experimental results have been discussed under three possible modes of analyte interaction (Figure 10) as follows:

### 3.1. Analyte –MWCNTs Interaction

During the analyte vapor exposure to the sensing device, the interaction between analyte - MWCNTs is expected to be predominant, as MWCNTs in this study have been dispersed almost uniformly over the Nylon-6 nanofibers. As known, analyte interaction on CNTs shifts the Fermi-level of CNTs [46, 47], which in turn leads to charge transfer. Our experimental study has shown that a MWCNTs/Nylon-6 nanofiber sensor has the highest responsiveness towards acetone (Table 1) whereas it was about 6-fold lower for EA. The dipole moment of the acetone molecule is larger than that of EA in this case the higher responsiveness could be attributed to this fact The study has demonstrated that DCM has about 10 times higher responsiveness than TCM, which is also ascribed to the larger dipole moment of DCM. Analyte molecules that possess electron withdrawing groups such as chlorine and carbonyl groups could exercise an electron pulling effect on the MWCNTs during the exposure, which leads for higher responsiveness. In particular, the carbonyl (C=O) group pulls electrons strongly from the MWCNTs due to its strong electron withdrawing nature and hence the highest responsiveness has been observed for acetone. The study has also shown that the lowest responsiveness among the polar solvents studied here was for THF (Table 1). The analyte THF, an electron donor in nature, could be involved in charge transfer to MWCNTs through its electron rich oxygen atom. The *sa* MWCNTs/Nylon-6 nanofiber based sensor exhibited the smaller response towards the hexane and toluene, which are apolar in nature. These results suggest that the nature of the analyte plays a vital role in controlling the performance of the *sa* MWCNTs/Nylon-6 nanofiber composite sensor. Our further analysis of the physical properties of analytes has hinted that analyte volatility also contributes to the differences in the responsiveness. The vapor pressure, a good indicator of solvent volatility, can be defined here as the condition required to become vapor from the container to the sensing zone in sensor. According to the National Technical Information Service (NTIS), any chemical with a vapor pressure of greater than 13.33 kPa (>100 mm Hg) is said to have high volatility. The vapor pressure of EA is *ca.* three times lower than acetone (Table 1), and likewise, the vapor pressure of TCM is two fold smaller than that of DCM at 25°C, indicating that DCM and acetone are more volatile at room temperature. Therefore at the room temperature (25°C), it can be expected that a larger number of acetone and DCM molecules arrive at the *sa* MWCNTs/Nylon-6 nanofibers mat, interact and thus are involved in charge transfer from MWCNTs. This is evident in the MWCNTs/Nylon-6 nanofibers based device's higher responsiveness towards acetone and DCM. Similarly, the vapour pressure of THF is much lower than the other analytes, and hence this could be another parameter that accounts for the device's poor responsiveness to this susbtance. Therefore, given the response time interval of 5 min and at 25°C, the magnitude of responsiveness has been found to be dependent on the dipole moment, vapour pressure and nature of polar functional groups of analytes.

### 3.2. Analyte - Nylon-6 Interaction

Interaction between Nylon-6 and analyte molecules are also equally possible and this occurs especially in a region where poor dispersion of MWCNTs exists. Swelling is a common phenomenon that is encountered when a polymer is exposed to strong polar analytes [23, 48]. Santos *et al.* [49] have remarked that well-defined but conducting nanofibers allow for easier electron transport upon undergoing extensive swelling. During the analyte-to-Nylon-6 interaction, the cross-sectional area (*A*) of the composite matrix is expected to increase due to swelling, which results in lower resistance (i.e. higher conductance) according to the following correlation:

(2) $$R=\rho \frac{L}{A}$$

where *L* is the thickness of the conducting matrix and *ρ* is the resistivity in Ohm-m.

Since the Nylon-6 polymer is an insulator, the conductivity in the *sa* MWCNTs/Nylon-6 composite sensor only arises from the MWCNTs and hence, the conductivity of the Nylon-6 composite depends on the interconnectivity of MWCNTs. Swelling of the polymer matrix increases the distance between the MWCNTs, thus loosing their interconnectivity that eventually leads to a decrease in the conductivity. This could also account for the larger increase in responsiveness of the *sa* MWCNT composite towards DCM, TCM and acetone.

**Figure 11.**
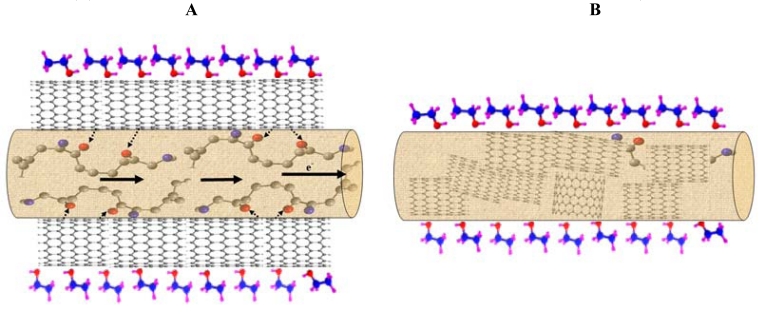
Schematic representation of (A) *sa* MWCNTs/Nylon-6 nanofibers composite and (B) *im* MWCNTs/Nylon-6 nanofibers during the exposure to an analyte (ethanol)

**Figure 12.**
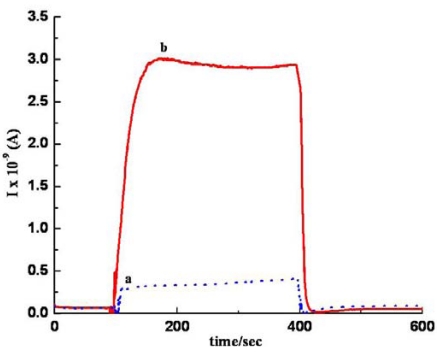
Response graph of (a) *im* MWCNTs/Nylon-6 nanofiber composite; (b) *sa* MWCNTs/Nylon-6 nanofiber composite during the exposure to ethanol at room temperature.

As another part of our study, the sensing performance of the *im* MWCNT/Nylon-6 nanofiber composite sensor has been investigated by exposing it to ethanol and comparing the results seen with the *sa* MWCNTs/Nylon-6 nanofiber based sensor. Given the identical analysis time, temperature and the same concentration of MWCNTs and ethanol analyte, the responsiveness of the *im* MWCNTs/Nylon-6 nanofibers has been found to be much smaller than that of the *sa* MWCNTs/Nylon-6 nanofiber based device (Figure 11). The analyte interaction with the MWCNT is weaker or even prevented in the case of the *sa* MWCNTs/Nylon-6 based sensor as the MWCNTs are infused in the Nylon-6 nanofibers, which could accounted for its poor gas responsiveness (Figure 12). This comparative study has suggested that the analyte-to-MWCNT interaction contributes significantly more to sensing performance than the analyte-to-Nylon-6 interaction. It is also to be noted that without Triton^®^ X-100 surfactant the MWCNTs in *im* MWCNT/Nylon-6 nanofibers have been found more aggregated (Figure 6) due to the van der Waals interactions between them. This could also be a reason for for the poor responsiveness of the *im* MWCNT/Nylon-6 nanofiber based device.

## 4. Conclusions

The feasibility of using *sa* MWCNTs/Nylon-6 nanofiber materials for gas sensing at room temperature has been demonstrated. The nanofiber based device has shown the significant reproducibility and responsiveness to organic analytes, especially to polar and non-polar molecules. It is expected that polar molecules with a carbonyl group might withdraw electrons from MWCNTs and hence the *sa* MWCNTs/Nylon-6 nanofiber composite has shown higher responsiveness towards acetone, compared to non polar molecules without those groups, like hexane and toluene. Overall, the dipole moment, nature of functional groups and vapor pressure of analytes are the key factors that influence the responsiveness of sensor device at room temperature. The *im* MWCNTs/Nylon-6 nanofiber based sensor has produced lower responsiveness compared to a *sa* MWCNTs/Nylon-6 nanofiber composite, suggesting that the analyte interaction with the MWCNT is important for increased responsiveness. Currently, our research group has been investigating the effects of temperature and analyte concentration to gain a better perspective of the wider applications of the *sa* MWCNTs/electrospun Nylon-6 composite. Recently, there has been growing interest in adopting well-aligned nanofiber materials for fabricating electrode systems in sensor devices [50, 51] to increase the sensitivity. It is expected that the *aligned* Nylon-6 nanofibers in the sensing device could allow for maximum dispersion of MWCNTs, thereby resulting in enhancement in the sensitivity, which is also under investigation in our laboratory.

## Figures and Tables

**Table 1.** Performance of *sa* MWCNTs/Nylon-6 nanofiber composite towards the various types of analytes.

**Analytes**	**Nature of Analytes**	**Dipole moment (D)**	**Vapor pressure kPa @ 25°C**	**Responsiveness**$\frac{R-R_{0}}{R_{0}}$

Acetone	Polar	2.91	30.8	28.70
Ethyl acetate (EA)		1.88	10.13	4.90
Dichloromethane (DCM)		1.60	58.2	11.68
Trichloromethane (TCM)		1.08	26.2	1.48
Tetrahydrofuran (THF)		1.63	21.6	1.38
Ethanol		1.60	7.87	4.34

Hexane	Non polar	0.08	20.2	3.55
Toluene		0.31	3.79	0.13
